# Clinical Profile of Polycystic Ovarian Syndrome in Women From Bhimavaram: A Cross-Sectional Study

**DOI:** 10.7759/cureus.81854

**Published:** 2025-04-07

**Authors:** Sri Chandana Mavulati, Sujatha Dodoala

**Affiliations:** 1 Pharmacology, Shri Vishnu College of Pharmacy, Bhimavaram, IND; 2 Pharmacology, Institute of Pharmaceutical Technology, Sri Padmavathi Mahila Visvavidyalayam, Tirupati, IND

**Keywords:** cyst, endometrium, follicles, phenotype, polycystic ovarian syndrome (pcos)

## Abstract

Introduction

Polycystic ovarian syndrome (PCOS) is a complex endocrinological dysfunction that affects the hypothalamic-pituitary-ovarian axis. Despite its high prevalence, variations in phenotypic presentation and associated risk factors remain underexplored. The study aims to identify the prevalence, biochemical, clinical, and hormonal features of PCOS women, emphasizing phenotype classification and therapeutic interventions.

Methods

This cross-sectional, prospective multi-centre study was conducted in outpatient gynaecology clinics in Bhimavaram, India, from July 2019 to December 2023. Participants were diagnosed based on the Rotterdam criteria and classified into phenotypes A, B, C, and D. Data on socio-demographics, clinical manifestations, hormonal profiles, and treatment adherence were collected and analysed using Epi Info (version 7.2.6.0; Centers for Disease Control and Prevention, Atlanta, GA). Associations between phenotypes and metabolic profiles were assessed using analysis of variance (ANOVA) and chi-square tests.

Results

Among the study population, 403 (8.02%) were diagnosed with PCOS, with phenotype C in 139 (40.76%) being the most prevalent, followed by phenotypes D (n=90, 26.39%), A (n=89, 26.10%), and B (n=23, 6.74%), respectively. Oligomenorrhoea was a frequent menstrual disturbance found in 145 women (42.52%). Acne was experienced by 191 participants (56.01%), and hirsutism was seen in 182 (53.32%), signifying them as common hyperandrogenic symptoms. It is also noted that a significant proportion of the study population was overweight or obese (n=162, 47.5%). Anaemia was observed in 268 participants (78.6%), while 71 (20.82%) women had hypothyroidism, and 14 (4.11%) were diabetic. Therapeutic management included clomiphene citrate, hormonal contraceptives, and metformin. However, adherence remained a key challenge in achieving therapeutic success.

Conclusion

With the increase in the prevalence rate, early diagnosis and lifestyle modifications are required to prevent long-term complications in women. Additionally, risk factors identified in this study can be modified with appropriate interventions and preventive measures.

## Introduction

Polycystic ovarian syndrome (PCOS) is a hormonal disorder that affects women of reproductive age, with a prevalence ranging between 5% and 20% globally [1-7]. This variation in prevalence is due to disparities in diagnostic standards, genetic background, and environmental factors such as body mass index and stress [7,8]. Endocrine disorder affects the hypothalamic-pituitary-ovarian axis, presenting different clinical manifestations such as menstrual irregularities, hyperandrogenism, and polycystic ovarian morphology [2-7]. These features often lead to significant comorbidities, such as metabolic syndrome, heart disease, and psychological disorders [7-9]. The heterogeneous characteristics of disorder have prompted many classification criteria; however, the Rotterdam criteria still remain as a standard method widely used by gynaecologists to diagnose PCOS.

The characteristics of PCOS can differ significantly among individuals, leading to the identification of distinct phenotypes: the classic phenotype (oligomenorrhea, hyperandrogenism, and polycystic ovaries), along with other types that may present without overt hyperandrogenism or irregular menstrual cycles [9,10]. The Rotterdam criteria are the internationally endorsed diagnostic category; the phenotypic approach of screening would help in recognizing women at high risk of metabolic dysfunctions such as dyslipidemia, cardiovascular diseases, thyroid issues, and increased glucose levels. Elevated glucose levels or imbalance of thyroid hormones interfere with granulosa cell function, which in turn affects the follicular development and ovulation, leading to infertility caused by PCOS [3,11]. Family history plays a crucial role, with studies indicating a higher incidence of PCOS among immediate family members. Obesity is another significant risk factor as it can exacerbate insulin resistance, a common metabolic disturbance in PCOS [9,10]. Though many published articles examined the frequency and common clinical profile in the various geographical areas, research that addresses phenotypic prevalence and its associated risk factors remains inconclusive.

Another important aspect of PCOS is its impact on the endometrium, the lining of the uterus. According to several studies, endometrial thickness may be associated with anovulation and altered hormonal profiles, potentially leading to challenges in conception [12]. Cyst type is another critical factor in understanding PCOS. The existence of several tiny follicles, often termed "cysts," can be indicative of the condition. However, not all cysts have the same implications for fertility. For instance, compared to other types of cysts, the hormonal activity and ovulation-related consequences of the functional ovarian cysts commonly found in PCOS may vary [13].

Treatment for managing PCOS often requires a collaborative approach that focuses on both the reproductive and metabolic aspects of the syndrome. Therapeutic options may include hormonal contraceptives to regulate menstrual cycles and manage hyperandrogenic symptoms, insulin sensitizers to improve metabolic health, and lifestyle interventions aimed at weight management [14,15]. Surgical therapy, such as laparoscopic ovarian surgery and in vitro fertilization (IVF), was found to alternative in case of therapeutic failure or women at high-risk of multiple pregnancies and severe ovulatory dysfunction [16]. Despite a wide range of drug options, the disorder was found to be incurable, and therapy is used to manage the symptoms. A thorough understanding of individual patient needs is essential for optimizing treatment plans. In conclusion, given the complexity of PCOS, a comprehensive knowledge of its phenotypes, clinical characteristics, risk factors, and prescribing patterns is critical for effective management. Continued research is necessary to refine treatment strategies and improve outcomes for affected women.

## Materials and methods

Study design and ethical considerations

An observational, cross-sectional study with a prospective design was carried out in an outpatient setting of the Gynaecology and Infertility Department in various clinics of Bhimavaram, Andhra Pradesh, India, from July 2019 to December 2023. The Institutional Ethical Committee of the Shri Vishnu Educational Society has approved the study (IEC Ref No: VDC/IEC/fac/2018/14).

Study sample

Of the 5,021 women visiting the clinics, 403 subjects were diagnosed with a confirmatory characteristic of PCOS. However, after the due explanation about the research objectives and protocol to the study participants, only 378 women showed willingness to participate in the cross-sectional research. After the initial screening, participants were informed about the trial objectives, purpose, and procedure. Written Informed consent was taken from all the patients stating that their involvement was voluntary, and confidentiality was maintained throughout the study.

Inclusion criteria

The participants included in the study were required to satisfy at least two of the diagnostic criteria according to the Rotterdam principle: (a) visualization of the polycystic ovaries in ultrasound, (b) symptoms associated with hyperandrogenism or increased serum testosterone levels, and (c) signs of oligomenorrhea or amenorrhea for six months [17].

Exclusion criteria

Patients with menarche of less than two years from the date of study due to immature hypothalamic-pituitary-ovarian axis development, ages under 15 years and surpassing 45 years, symptomatic diseases of the liver, kidney, heart, congenital hyperplasia, androgen-secreting tumours, and those who declined to take part in the study were excluded. Further, 37 respondents' data were excluded because they provided incomplete or partial information.

Data collection

A questionnaire was designed and used to gather the patient’s medical and drug history. The data collected includes socio-demographic characteristics, such as age, weight, age at menarche, length of the period cycle, marital status, and education. The World Health Organization recommendations were used to assess body mass index: underweight (<18 kg/m^2^), normal weight (18-24.9 kg/m^2^), overweight (25-29.9 kg/m^2^), and obese (>30 kg/m^2^) [18]. Family history of disorders such as PCOS, infertility, diabetes, and thyroid was noted. Women were further categorized as newly diagnosed, diagnosed, under treatment, discontinued, and revisited to understand their adherence patterns. Data on sleep disturbances, exercise, diet, and eating habits were collected to evaluate lifestyle habits.

Clinical examination

Data on ultrasound findings of the ovaries were obtained. Polycystic ovaries are characterized by the presence of follicles (greater than or equal to 12) ranging from 2 to 9 mm in size, with or without ovarian volume above 10 mL. Patients experiencing painful periods were classified as having dysmenorrhea, and those with an absence of periods for more than three cycles as amenorrhea. Oligomenorrhea women experience a delay in menstruation >35 days to six months, whereas menorrhagia is heavy or prolonged menstrual bleeding. Endometrium thickness was noted to understand menstrual phases. The presence of excess androgen secretion was explained by the Ferriman-Galloway (FG) score, which assesses hirsutism across nine body regions, assigning scores ranging from 0 to 4 in the areas of upper lip, chin, chest, abdomen (upper and lower), thighs, upper limb, and upper and lower back. Acne was assessed based on the Global Acne Grading System by identifying no lesions as Grade O and increasing the severity based on the presence of comedones, papules, pustules, and nodules in the chin, nose, forehead, right and left cheek, chest, and upper back [8].

Study participants’ current and past histories of diseases, such as thyroid and diabetic profiles, were gathered. The hormonal status was assessed to determine the LH/FSH ratio. A thyroid profile TSH > 4.5 mIU/L was considered hypothyroid. Diabetic levels were analysed using fasting blood glucose values and classified as hypoglycemic (less than 70 mg/dL), normal (between 70 and 99 mg/dL), pre-diabetic (ranges from 100 to 125 mg/dL), and diabetes (≥ 125 mg/dL).

Phenotype classification

Women were classified according to the National Institutes of Health (NIH, 2012) organized an evidence-based workshop on PCOS, where the experts reaffirmed the validity of the Rotterdam Criteria in 2003. They also identified specific sub-phenotypes within these criteria: (A) excessive androgen, ovulatory disturbances, and PCOM; (B) androgen excess associated with ovulatory dysfunction; (C) hyperandrogenism with polycystic ovarian morphology (PCOM); and (D) ovulatory dysfunction accompanied by PCOM [16]. The Rotterdam criteria were accepted and endorsed as widely used standard guidelines for the assessment of PCOS management. Finally, drug utilization patterns were examined to analyse trends based on clinical characteristics.

Data analysis

Summary statistics were described using average/mean and percentage for categorical data. The Epi Info software tool (version 7.2.6.0; Centers for Disease Control and Prevention, Atlanta, GA) is used for data analysis. Discrete variables of study were represented in terms of numerical values and percentages, whereas quantitative variables are mentioned as mean ± standard deviation. Appropriate significance tests were selected based on the characteristics and distribution of the variables, including the chi-square for qualitative variables. Analysis of variance (ANOVA) is used to analyse clinical parameters. Outcome significance was estimated at p < 0.05.

## Results

**Figure 1:**
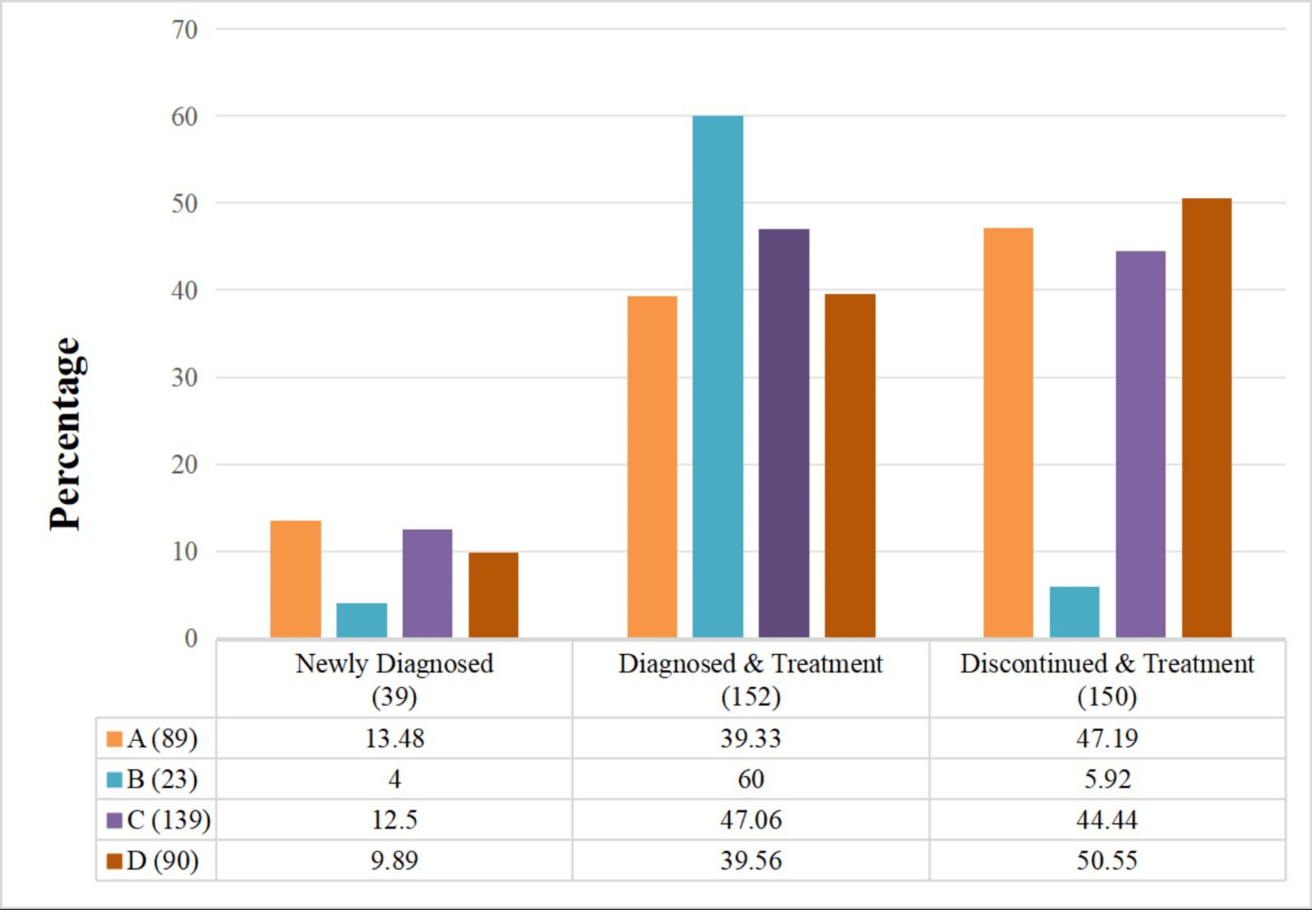
Distribution of PCOS phenotypes across diagnosis and treatment categories The bar graph illustrates the percentage distribution of four PCOS phenotypes: A (hyperandrogenism + ovulatory dysfunction + polycystic ovarian morphology), B (hyperandrogenism + ovulatory dysfunction), C (hyperandrogenism + polycystic ovarian morphology), and D (ovulatory dysfunction + polycystic ovarian morphology) across three clinical groups: newly diagnosed (n = 39), diagnosed & treatment (n = 152), and discontinued & treatment (n = 150). Each bar represents the percentage of individuals within a phenotype group for each clinical category. Colors used to differentiate phenotypes are A (orange), B (blue), C (purple), and D (brown). PCOS: Polycystic ovarian syndrome

PCOS is the most prevalent multifaceted metabolic disorder that requires therapeutic intervention from different clinicians to treat it. Owing to its heterogeneity, each subject requires specialized and individualized attention. The prevalence rate was observed at 403 (8.02%), and from the diagnosed cases, only 341 participants were enrolled; the remaining were excluded due to unwillingness and incomplete case profiles. From enrolled participants, 39 (11.43%) women were newly diagnosed, 152 (44.57%) were diagnosed prior and were under treatment, whereas 150 (43.99%) participants discontinued the regimen and revisited the clinic for therapy. Figure 1 displays the prevalence of PCOS phenotypes. Phenotype C is found in 139 (40.76%), exhibiting the highest prevalence, followed by Phenotype D in 90 (26.39%) and 89 (26.10%) people belonging to Phenotype A. Phenotype B was the least reported in the study population (23, 6.74%).

The patients' demographic profile is shown in Table 1. The study group's average age was found to be 26.84±5.60 years, with 210 (61.6%) of the population falling into the 21-30 age range. Menarche occurred at an average age of 12.8±0.8 years. Additionally, it was found that the majority of people (n=305, 89.44%) who came to the clinic were married, and infertility was their main issue (Table 1). Different phenotypes of PCOS were compared with clinical and hormonal parameters using the appropriate statistical test (ANOVA). The values are represented in Table 2, which shows that there are slight variations in clinical values across four phenotypes. The results of continuous data are represented as mean ± standard deviation, whereas categorical variables are expressed as n (%).

**Table 1:** Sociodemographic and anthropometric characteristics of the study participants BMI: Body mass index; values are expressed as n (%).

Parameters	Variables	Number of Participants (n=341) (%)
Age group(years)	≤20	45 (13.2)
	21-30	210 (61.6)
	31-40	79 (23.2)
	≥40	7 (2.05)
BMI (kg/m^2^)	Underweight (<18.5)	23 (6.74)
	Normal (18.5-24.9)	156 (45.75)
	Overweight (25-29.9)	117 (34.31)
	Obese (30- above 40)	45 (13.20)
Education	Primary	48 (14.08)
	Secondary	74 (21.70)
	Graduate	161 (47.21)
	Post Graduate	58 (17.01)
Marital status	Yes	305 (89.44)
	No	36 (10.56)

**Table 2:** Clinical and hormonal parameters across PCOS phenotypes BMI: Body mass index; Hb: Hemoglobin; LH: Luteinizing hormone; FSH: Follicle stimulating hormone; PCOS: Polycystic ovarian syndrome Cycle refers to the number of bleeding days during the menstrual cycle. Values are expressed as mean ± standard deviation (SD). Analysis: One-way ANOVA was used to compare clinical and hormonal parameters across PCOS phenotypes. ^*^Indicates statistically significant difference (p<0.05); ^†^ denotes highly significant difference (p<0.01).

Parameters	A	B	C	D	p-value
BMI (kg/m^2^)	25.28 ± 5.12	23.91 ± 3.94	25.12 ± 4.36	25.10 ± 4.01	0.58
Hb (g/dL)	10.79 ± 1.38	10.76 ± 1.10	10.92 ± 1.29	10.58 ± 1.30	0.28
Cycle (days)	4.86 ± 1.86	3.96 ± 1.09	4.61 ± 1.11	4.31 ± 1.35	0.001^†^
Endometrium thickness (mm)	9.13 ± 2.25	9.74 ± 2.38	8.77 ± 2.12	9.59 ± 1.95	0.01*
LH (IU/L)	11.73 ± 1.78	11.23 ± 1.82	11.43 ± 1.76	11.93 ± 1.94	0.52
FSH (IU/L)	5.58 ± 1.14	5.60 ± 1.11	5.60 ± 1.21	5.42 ± 1.07	0.70
Thyroid (mIU/L)	3.01 ± 1.73	2.77 ± 1.12	3.23 ± 1.75	3.29 ± 2.02	0.60
Blood sugar (mg/dL)	99.29 ± 15.38	104.4 ± 12.32	99.69 ± 17.65	98.27 ± 15.42	0.40

**Figure 2:**
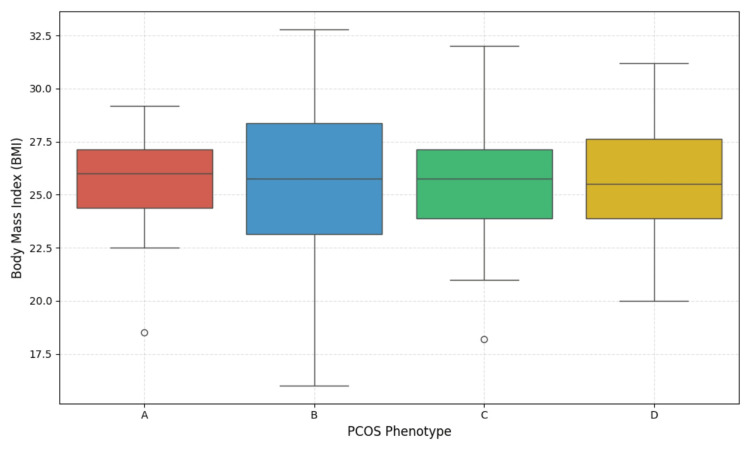
Body mass index (BMI) distribution among PCOS phenotypes The box plot shows BMI variation among the four PCOS phenotypes: A (ovulatory dysfunction + hyperandrogenism + polycystic ovarian morphology), B (ovulatory dysfunction + hyperandrogenism), C (hyperandrogenism + polycystic ovarian morphology), and D (ovulatory dysfunction + polycystic ovarian morphology). The X-axis represents PCOS phenotypes (A-D), and the Y-axis indicates BMI in kg/m^2^. Each box plot depicts the interquartile range (IQR), with the horizontal line representing the median. Whiskers indicate 1.5 x IQR, and individual dots denote outliers. PCOS: Polycystic ovarian syndrome

Figure 1 shows that Phenotype A exhibits slightly lower median BMI and Phenotype C has a higher median impact on value, referring to a link between phenotypes and metabolic risk. Meanwhile, Phenotype B did not have an impact on BMI (Figure 2). These findings suggest an association between phenotypes and comorbidity, so they clearly emphasize the need for an individualized therapeutic strategy. Oligomenorrhoea was the most common menstrual irregularity observed in 145 (42.52%) of the study population, followed by amenorrhoea in 71 (20.82%), as shown in Figure 1. Bilateral PCOS was observed in 181 (53.08%) women, whereas 72 (21.11%), 52 (15.24%), and 36 (10.56%) women in the study had a cyst on the right side, left side, and multiple follicles, respectively.

**Figure 3:**
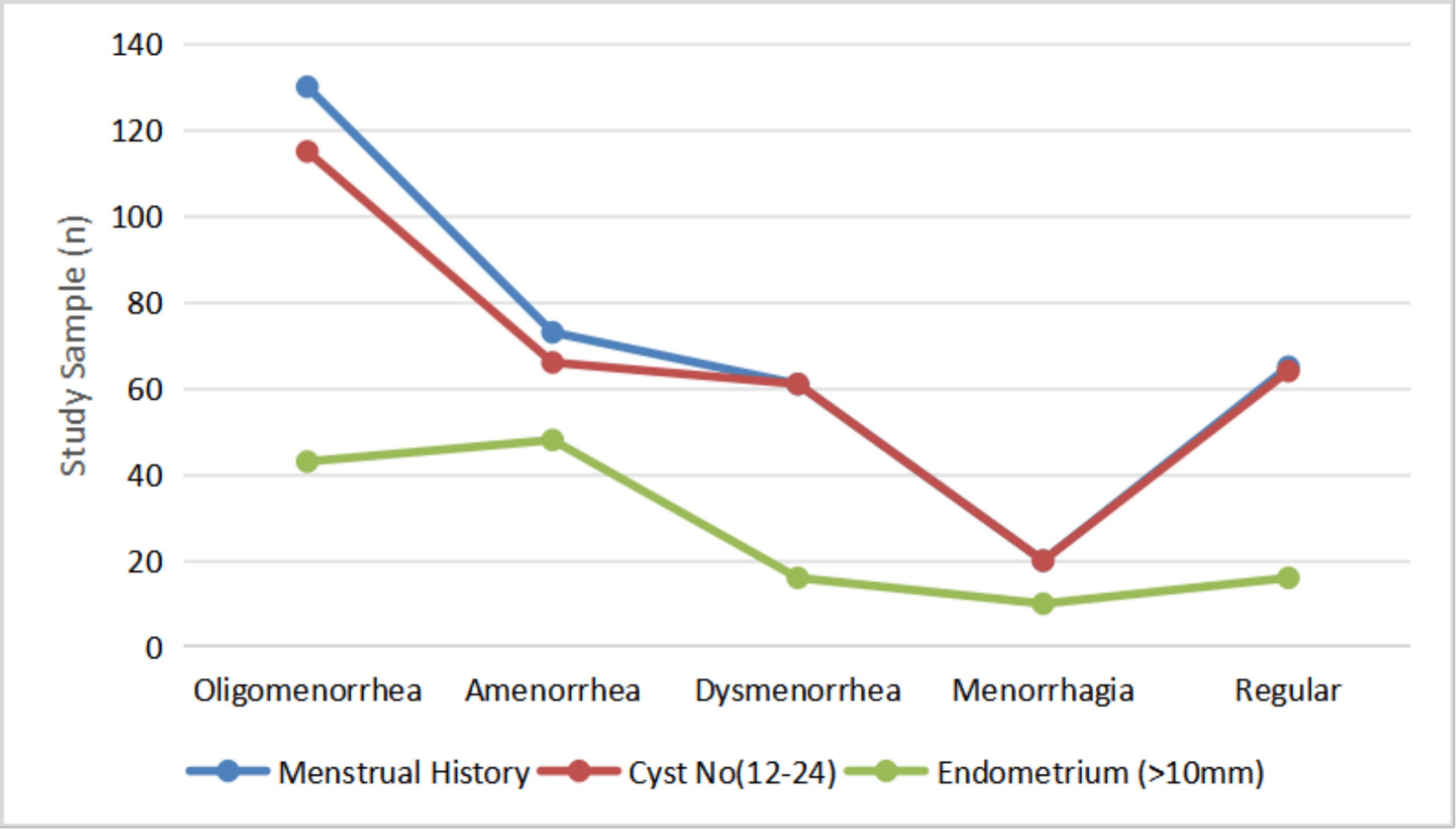
Frequency of women exhibiting variations in individual PCOS-related characteristics: menstrual history (blue), ovarian cyst count (12-24 follicles, red), and endometrial thickness >10 mm (green) across different menstrual conditions The X-axis represents different menstrual conditions: oligomenorrhea, amenorrhea, dysmenorrhea, menorrhagia, and regular cycles. Y-axis indicates the number of participants (n). Statistical significance was assessed using the chi-square test (p-value=0.018). Note: These clinical features represent individual diagnostic components of polycystic ovarian syndrome (PCOS) and do not indicate full phenotypes, which require a combination of two or more characteristics.

PCOS women experienced menstrual cycle bleeding at a mean of 4.55±1.42 days, and the average endometrium thickness was observed at 9.16±2.16. Figure 3 depicts the correlation of menstrual history with cyst number and endometrial thickness with a significant p-value (<0.05). Across different phenotypes, endometrium thickness had significant variation. It is observed that there was a significant variation in menstrual irregularities with relevance to hyperandrogenism, the presence of cysts, and endometrium thickness variation (chi-square test, p-value<0.05). The LH-FSH ratio in the study population was identified at 2:1 in all the phenotypes.

**Figure 4:**
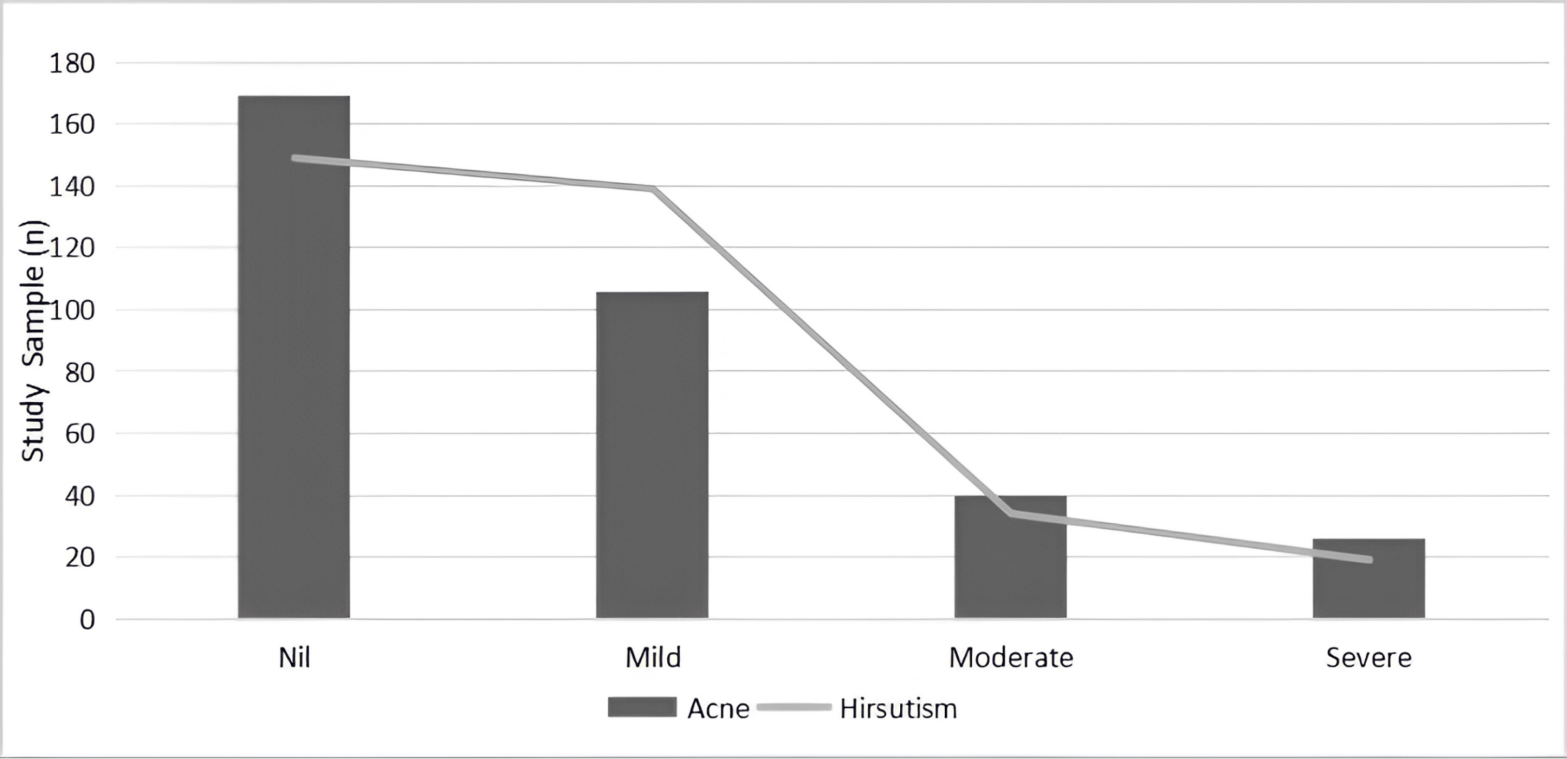
Comparison of acne (bar graph) and hirsutism (line plot) severity across clinical grades in polycystic ovarian syndrome (PCOS) participants The X-axis represents severity levels of clinical features: Nil (no symptoms), Mild, Moderate, and Severe. The Y-axis indicates the number of study participants (n). The bar graph illustrates the frequency of acne, while the line plot depicts the frequency of hirsutism across severity levels. Both acne and hirsutism are clinical indicators of hyperandrogenism, a key diagnostic component of PCOS phenotypes.

From the bar graph, it is observed that 182 (53.32%) subjects exhibited hirsutism, and 191 (56.01%) had signs of acne (Figure 4). Signifying hyperandrogenism in more than 50% of the population. The metabolic profile was assessed, in which 71 (20.82%) women had hypothyroidism, 14 (4.11%) were found to be diabetic, and 176 (51.61%) were at the pre-diabetic phase. Many of the women did not follow proper dietary habits and had never exercised. Hundred and thirty-three (39%) participants were found to eat fast food regularly, and sleep deprivation was observed in 186 (54.55%) women. Menstrual disturbances and lifestyle patterns have influenced their biochemical profile, where 268 (78.6%) women were mildly anaemic, followed by 24 (7%) and 17 (4.9%) being moderately and severely anaemic, respectively.

**Figure 5:**
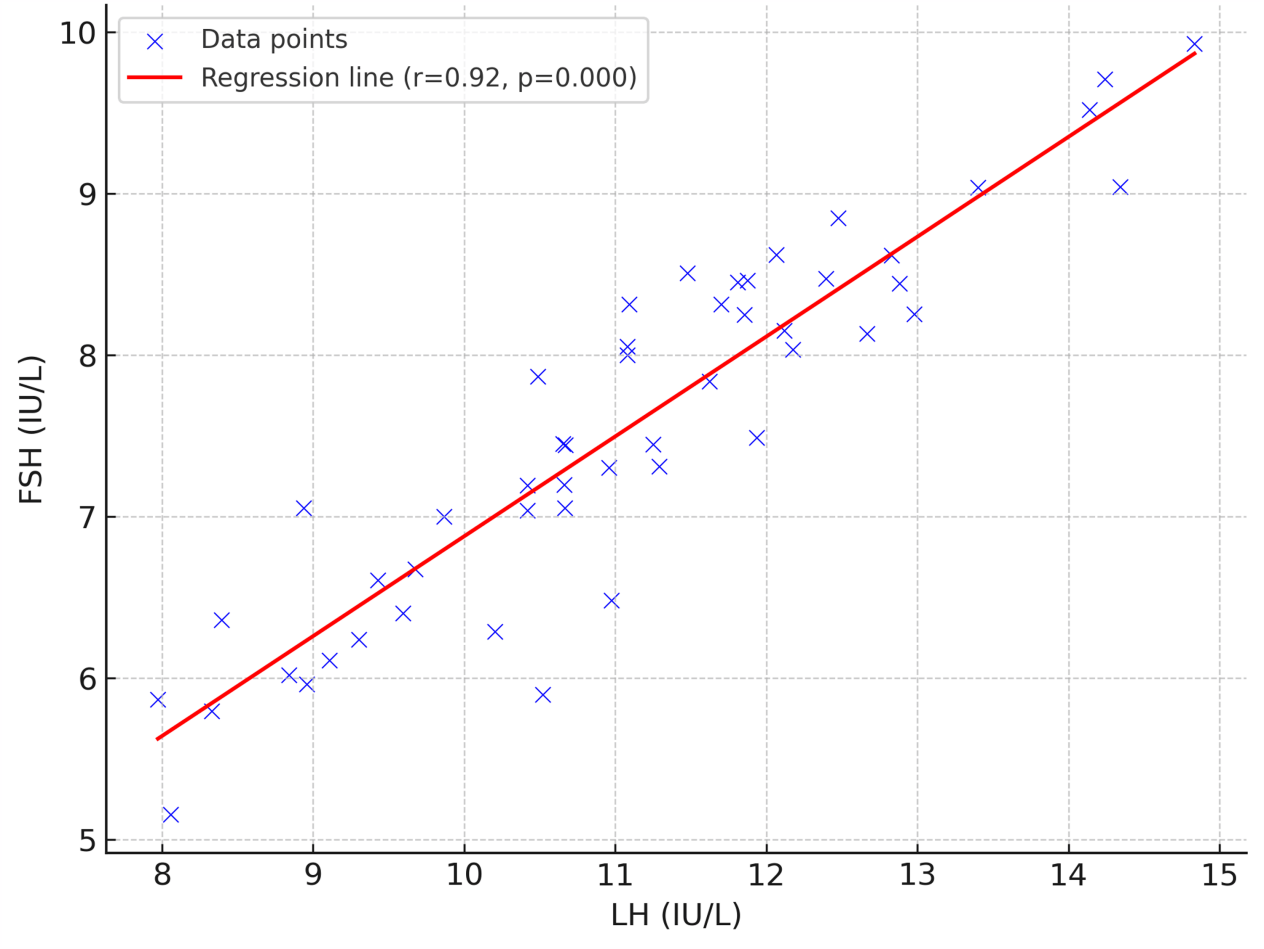
Correlation between LH and FSH levels in women with polycystic ovarian syndrome (PCOS) The X-axis represents the luteinizing hormone (LH) levels (IU/L), and the Y-axis represents follicle stimulating hormone (FSH) levels (IU/L). The scatter plot shows a strong positive correlation between LH and FSH (r = 0.92, p = 0.000), as indicated by the red regression line. This LH/FSH surge is a characteristic feature of hormonal imbalance commonly observed in women with polycystic ovarian syndrome (PCOS).

The scatter plot shows that the variables LH and FSH have a positive correlation (regression line, r=0.92, p<0.001) with one another. The plot's data points are dispersed throughout the regression line. When one variable rises, the other usually follows suit (Figure 5).

Table 3 represents the percentage of medication used by study participants, with the highest prescription frequency to clomiphene citrate for ovulation induction, followed by hormonal drug usage to regularize the cycles and hirsutism. Contraceptives and hormonal therapy were found to be common therapies in PCOS women; also, long-term and continuous therapy achieved good outcomes. However, it is observed that many participants reported an increase in weight, headache, flushing, discomfort in the lower abdomen, and nausea as some of the unwanted effects of the medication, which made them discontinue the drug. Thus, adherence to the prescription can be a significant challenge for PCOS women. Addressing the significance of therapy and supportive care would be crucial for enhancing the overall outcome of therapy. Engaging the patients with open discussions and counselling them regarding the unwanted effects of therapy would help the healthcare professionals to overcome the challenges.

**Table 3:** Therapeutic profile of women with polycystic ovarian syndrome (PCOS) presented as frequency and percentage (%) Values are presented as frequency and percentage (%). Others include myo-inositol, spironolactone, and antibiotics.  Inj HCG refers to injectable human chorionic gonadotropin. All participants reported adherence to lifestyle modifications as part of their therapeutic regimen.

Intervention	Frequency (%)
Lifestyle	341 (100)
Folic Acid/Multivitamins	225 (68.98)
Pain Killers	155 (45.45)
Clomiphene Citrate	130 (38.13)
Estradiol	120 (35.19)
Norethisterone	111 (32.55)
Medroxyprogesterone	64 (18.76)
Inj HCG	44 (12.9)
Combined Oral Contraceptives	42 (12.32)
Others	31 (9.1)

## Discussion

PCOS is the most prevalent reproductive disorder. Study observations align with the previous research emphasising individualized therapeutic strategies. The percentage of women with PCOS in India ranges from 3.7 to 22.5% [13], while our findings revealed a prevalence of 403 (8.02%). The distribution of participants reflects the chronic and relapsing nature of PCOS, stating the poor adherence rate to PCOS therapy. Furthermore, the phenotypic prevalence observed in the study aligns with the variability in clinical presentations reported by Ganie et al. [7]. Study reports emphasise that the majority of the population (210, 61.6%) belongs to the age group of 21-30 years, with an average age of 26.84±5.60 years, which is comparable to many studies with similar distribution in cohorts [6,19]. Additionally, the menarche age was identified at 12.8±0.8 years, and notably, 305 women (89.44%) of participants were married, whose major concern in visiting the clinic was infertility. Tay et al. identified similar reports stating that infertility is the primary reason for participants' clinic visits [9]. Weight aspects were found to affect the metabolic profile in women; in the study, it was observed that 162 women (47.51%) of the population were identified to be overweight or obese, raising the relationship between BMI and PCOS. Education background did not show much clinical significance; graduates presented a higher prevalence rate at 161 (47.21%). It is due to stress and lifestyle changes that they were prone to the condition, whereas some subjects were found to be less educated (n=122, 35.78%) and visited the clinics at later stages with more complications. Additionally, it is found that the statistical test ANOVA has shown significant differences within cycle (days) and endometrium thickness (mm) across phenotypes (p < 0.05). It is noted that all the phenotypes are exhibiting a prediabetic phase in Table 2, signifying the need for early management. Phenotype A population has an overweight BMI (25.28±5.12), whereas phenotype C shows slightly higher values than normal BMI (25.12±4.36). Thus, findings reveal that Phenotype variation is associated with metabolic risks.

Ethnic origin influences the complexity of symptoms; there is a greater need to know the characteristics of the same condition in different populations. It is a disorder that cannot be cured, but its symptoms can be managed. Therefore, treatment is performed by identifying the range of symptoms. Cysts were found in both ovaries of 181 women (53.08%) [13]; however, by observing each ovary for cysts, it was found that the probability of cyst formation was marginally greater in the right ovary (n=72, 21.11%) compared to the left ovary (n=52, 15.24%). Most of the women (145, 42.52%) had oligomenorrhoea, followed by amenorrhoea in 71 (20.82%). Previous studies have shown that menstrual cycle disturbances are one of the chief clinical characteristics of PCOS, which is the underlying cause of infertility.

According to the Rotterdam criteria, another clinical sign seen in PCOS is hyperandrogenism, which in our study population was represented based on hirsutism and acne. However, nearly 90 (27%) of the population did not exhibit the clinical signs. These findings are consistent with research by Mandrelle et al. [20] and Alakananda et al. [21], which documented hyperandrogenism prevalence exceeding 50% in the PCOS population. Kar conducted a study and found that menstrual dysfunction and androgen excess significantly have an impact on the quality of life. Metabolic disturbances were noted with thyroid and glucose level discrepancies in the study population, which echoes the findings of Kar [22]. Results also highlighted that most of the women were anaemic (268, 78.6%) due to heavy bleeding and menstrual irregularities.

The current study is the first to provide information on the phenotype prevalence in Bhimavaram (South India). PCOS is a disorder that results in long-term complications, and many studies suggest that diagnosis with phenotype classification would help counsel women about future risks. Most of the women (n=139, 40.76%) in the study belonged to Phenotypes C (excess androgen secretion and cystic ovaries), D (cystic ovaries and ovulatory dysfunction) (n=90, 26.39%), and A (89, 26.10%; ovulatory dysfunction, hyperandrogenism, and cystic ovaries). With variation in phenotype, the risk of comorbidity increases. Our analysis indicates that Phenotypes C, B, and A exhibit a higher mean BMI (above 25), leaning towards the overweight category, compared to Phenotype B. The findings also indicate that the aforementioned phenotypes are associated with greater severity of PCOS, as evidenced by increased endometrial thickness and follicle count, particularly in Phenotype A (15.83±2.08) and Phenotype D (15.07±2.75), with significance at p-value <0.05. Therefore, to prevent metabolic risk, women should undergo frequent diagnoses of thyroid and glucose levels.

The study results show that BMI, hormonal discrepancies (thyroid and insulin), and genetic profiles with metabolic or hormonal disorders were found to be predisposing factors of PCOS, which is accepted by other authors [18,20]. Lifestyle changes are regarded as the primary approach for managing individuals with PCOS, particularly for those who are overweight and obese. In the case of drug therapy, metformin and clomiphene citrate were considered as first-line treatment [23]. Hormonal (320, 93.84%) and vitamin (n=228, 68.98%) intake was the highest in the study population. However, oral contraceptive pills were found to be the better option for treating menstrual irregularities in the case of unmarried girls and recently married women [24]. For fertility issues, clomiphene citrate was administered at standard dosage, and the dosage was increased as needed or combined with gonadotropins [25]. It is observed that ovulation was induced with a single dose of human chorionic gonadotropins (10,000 IU) once the follicle size and endometrial thickness were optimal. Study participants experienced some unwanted effects such as lower abdominal discomfort, headache, flushing, nausea, and weight gain [26].

Finally, the study reinforces the multifaceted profile of PCOS, which requires the regular monitoring of metabolic and hormonal parameters to mitigate long-term complications. Future research should focus on exploring personalised treatment approaches and strategies to enhance adherence to therapy.

Limitations of the study

The current research study has a few limitations. The study was conducted in an outpatient department of gynaecological hospitals in Bhimavaram, which limits the applicability of results to diverse populations. However, we have conducted the study in a multi-centre at Bhimavaram for assessing the clinical profile and treatment pattern of multiple clinicians.

## Conclusions

In the current study, the regional prevalence was observed at 403 (8.02%), and there is a significant association between phenotype and reproductive profile. Most of the study participants exhibited hyperandrogenism and hormonal discrepancies. Hormonal drugs, clomiphene citrate, and metformin were the first-line drugs used primarily in the disease population. Many women did not follow the proper lifestyle pattern and were found to discontinue the treatment, which is the key issue, resulting in therapeutic failure and increasing the risk of metabolic disturbances. Future investigations are required to address the following research questions and should also explore the strategies to enhance adherence to therapy in PCOS women. Furthermore, there is a need for healthcare professionals to understand the psychological factors responsible for distress and discontinuation of therapy, which would ensure effective therapeutic management.

## References

[1] Bharali MD, Rajendran R, Goswami J, Singal K, Rajendran V (2022). Prevalence of polycystic ovarian syndrome in India: a systematic review and meta-analysis. Cureus.

[2] Skiba MA, Islam RM, Bell RJ, Davis SR (2018). Understanding variation in prevalence estimates of polycystic ovary syndrome: a systematic review and meta-analysis. Hum Reprod Update.

[3] Farhadi-Azar M, Behboudi-Gandevani S, Rahmati M, Mahboobifard F, Khalili Pouya E, Ramezani Tehrani F, Azizi F (2022). The prevalence of polycystic ovary syndrome, its phenotypes and cardio-metabolic features in a community sample of Iranian population: Tehran lipid and glucose study. Front Endocrinol (Lausanne).

[4] Joshi B, Mukherjee S, Patil A, Purandare A, Chauhan S, Vaidya R (2014). A cross-sectional study of polycystic ovarian syndrome among adolescent and young girls in Mumbai, India. Indian J Endocrinol Metab.

[5] Gupta M, Singh D, Toppo M, Priya A, Sethia S, Gupta P (2017). A cross sectional study of polycystic ovarian syndrome among young women in Bhopal, Central India. Int J Community Med Public Health.

[6] Gill H, Tiwari P, Dabadghao P (2012). Prevalence of polycystic ovary syndrome in young women from North India: a community-based study. Indian J Endocrinol Metab.

[7] Ganie MA, Chowdhury S, Malhotra N (2024). Prevalence, phenotypes, and comorbidities of polycystic ovary syndrome among Indian women. JAMA Netw Open.

[8] Tay CT, Hart RJ, Hickey M (2020). Updated adolescent diagnostic criteria for polycystic ovary syndrome: impact on prevalence and longitudinal body mass index trajectories from birth to adulthood. BMC Med.

[9] Tay CT, Loxton D, Bahri Khomami M, Teede H, Harrison CL, Joham AE (2023). High prevalence of medical conditions and unhealthy lifestyle behaviours in women with PCOS during preconception: findings from the Australian Longitudinal Study on Women's Health. Hum Reprod.

[10] Begum G, Shariff A, Ayman G, Mohammad B, Housam R, Khaled N (2017). Assessment of risk factors for development of polycystic ovarian syndrome. Int J Contemp Med Res.

[11] El-Mazny A, Abou-Salem N, El-Sherbiny W, El-Mazny A (2010). Insulin resistance, dyslipidemia, and metabolic syndrome in women with polycystic ovary syndrome. Int J Gynaecol Obstet.

[12] Shah B, Parnell L, Milla S, Kessler M, David R (2010). Endometrial thickness, uterine, and ovarian ultrasonographic features in adolescents with polycystic ovarian syndrome. J Pediatr Adolesc Gynecol.

[13] Sonak M, Rathod PD, Patankar US (2022). A prospective observational study of polycystic ovarian syndrome among adolescent and young girls at tertiary care hospital. Int J Reprod Contracept Obstet Gyneco.

[14] Tanbo T, Mellembakken J, Bjercke S, Ring E, Åbyholm T, Fedorcsak P (2018). Ovulation induction in polycystic ovary syndrome. Acta Obstet Gynecol Scand.

[15] Costello MF, Misso ML, Wong J (2012). The treatment of infertility in polycystic ovary syndrome: a brief update. Aust N Z J Obstet Gynaecol.

[16] Teede HJ, Misso ML, Costello MF (2018). Recommendations from the international evidence-based guideline for the assessment and management of polycystic ovary syndrome. Fertil Steril.

[17] McCook JG, Reame NE, Thatcher SS (2005). Health-related quality of life issues in women with polycystic ovary syndrome. J Obstet Gynecol Neonatal Nurs.

[18] Kivimäki M, Strandberg T, Pentti J (2022). Body-mass index and risk of obesity-related complex multimorbidity: an observational multicohort study. Lancet Diabetes Endocrinol.

[19] March WA, Moore VM, Willson KJ, Phillips DI, Norman RJ, Davies MJ (2010). The prevalence of polycystic ovary syndrome in a community sample assessed under contrasting diagnostic criteria. Hum Reprod.

[20] Mandrelle K, Kamath MS, Bondu DJ, Chandy A, Aleyamma T, George K (2012). Prevalence of metabolic syndrome in women with polycystic ovary syndrome attending an infertility clinic in a tertiary care hospital in South India. J Hum Reprod Sci.

[21] Alakananda A, Das BP, Goel I (2017). A study on clinical profile of patients with polycystic ovarian syndrome. Int J Sci Res.

[22] Kar S (2013). Anthropometric, clinical, and metabolic comparisons of the four Rotterdam PCOS phenotypes: a prospective study of PCOS women. J Hum Reprod Sci.

[23] Kar S, Sanchita S (2015). Clomiphene citrate, metformin or a combination of both as the first line ovulation induction drug for Asian Indian women with polycystic ovarian syndrome: a randomized controlled trial. J Hum Reprod Sci.

[24] Yu JH, Moon MK, Ahn HC, Yang YM (2024). Assessing medication use patterns among patients with polycystic ovary syndrome at a tertiary care teaching hospital in South Korea: a retrospective study. Medicine (Baltimore).

[25] Weiss NS, Kostova E, Nahuis M, Mol BW, van der Veen F, van Wely M (2019). Gonadotrophins for ovulation induction in women with polycystic ovary syndrome. Cochrane Database Syst Rev.

[26] Rashid R, Mir SA, Kareem O (2022). Polycystic ovarian syndrome-current pharmacotherapy and clinical implications. Taiwan J Obstet Gynecol.

